# The origins of language in teaching

**DOI:** 10.3758/s13423-016-1077-7

**Published:** 2016-07-01

**Authors:** Kevin N. Laland

**Affiliations:** Centre for Social Learning and Cognitive Evolution, School of Biology, University of St Andrews, Harold Mitchell Building, St Andrews, Fife, KY16 9TH Scotland, UK

**Keywords:** Language, Teaching, Evolution

## Abstract

I introduce seven criteria for determining the validity of competing theories for the original function of language. I go on to present a novel explanation that meets all the criteria: language originally evolved to teach kin. I suggest that the use of symbols subsequently generated evolutionary feedback at two levels, in the form of self-modified selection pressures that favored structures in the mind that functioned to manipulate and use symbols with efficiency, and cultural selection on languages for learnability.

Other apes do not have functionally referential alarm calls (Hauser, 1996), and their gestures are neither deployed referentially nor exhibit symbolic or conventionalized features (Pika et al., 2005). The evolution of language seemingly required a shift in referential communication away from specific isolated unlearned signals applied by animals to concrete events in the present to a general, flexible, learned and socially transmitted, infinitely combinable, and functionally unconstrained form of communication; something entirely absent in the animal world (Fitch, 2010; Hurford, 2014).

Selective scenarios for how and why this transition to the emergence of natural language occurred are bounteous: language evolved to facilitate cooperative hunting (Washburn & Lancaster, 1968), as a substitute for grooming (Dunbar, 1998), to promote pair bonding (Deacon, 2003), to gossip about others (Power, 1998), as a tool for thought (Burling, 1993), or to fulfill countless other functions or purposes. The wealth of diverse historical narratives is enough to leave some researchers deeply sceptical as to the value of such theorizing (Hauser et al., 2014).

Moreover, human language exhibits a multitude of distinct functions, implying that, for whatever reason complex language originally evolved, it would have been coopted for uses unconnected to its original function. Distinguishing between a genuine selective scenario and the subsequent exploitation of what is surely one of human beings’ most flexible characteristics is extremely difficult. Nonetheless, criteria can be deployed that allow researchers to judge the relative merits of alternative historical narratives on the original function of language, but to my knowledge such criteria have never been fully compiled, and have not been applied in unison. The approach that I follow was pioneered by Szamado & Szathmary (2006) and Bickerton (2009), who between them sketched six criteria for determining the validity of competing language evolution theories. To these I add one further criterion of my own (Odling-Smee & Laland, 2009), to generate seven benchmarks with which to evaluate alternative explanations for the original adaptive advantage conferred by early forms of language. This compilation of criteria is important because, although individually most of the criteria are not particularly constraining, taken together they comprise a tough standard against which almost all of the proposed accounts fail.

These seven constraints are: (1) *The theory must account for the honesty of early language*. Human language constitutes a uniquely cheap and flexible signalling device, allowing humans to engage in ‘cheap talk’ in an unprecedented range of circumstances. However, if words are easy and cost-free to produce, why should anyone believe what others say, and what is the incentive to learn thousands of words if one cannot be confident that any convey an accurate message? This constraint implies that researchers should favor theories that propose a context for the evolution of early language in which there was either no conflict of interest between the signaller and receiver, or one in which the reliability of the signals could easily be assessed (Szamado & Szathmary, 2006). (2) *The theory should account for the cooperativeness of early language*. In many acts of linguistic communication, the transmitter imparts information that benefits the receiver, raising the issue of what is in it for the transmitter. The successful theory must explain why, at the time of the origins of language, an individual would go out of its way to help another individual by passing on information. (3) *The theory should explain how early languages could have been adaptive from the outset*. Bickerton frames this constraint in the form of a ‘ten word test’: a challenge for any account of language evolution is to explain what could usefully be said in just a few words. (4) *The theory should explain symbol grounding*. There has to some means by which early words could acquire their meaning, for instance, through pointing, imitation, or some form of representation. (5) *The theory should explain the generality of language*. Language is characterized by the range and power of generalization that it confers. Humans can transmit information about the past and the future, as well as events or objects distant in space. (6) *The theory should account for the uniqueness of human language*. A compelling theory of language evolution must explain why the context that favored language in humans either did not arise, or did not favor the evolution of language, in any other species (Hurford, 1999). (7) *The theory should explain why communication needed to be learned*. Leaving aside the role of evolved structure in language acquisition, human language is learned socially. Given that non-human primate communication is largely unlearned (Janik & Slater, 1997; Fitch, 2010; Hurford, 2014), and changes at rates little different from other biologically evolved characters, the question arises: what was language needed for, that required it to be both socially learned and rapidly changing? Theory suggests that cultural transmission is favored in changeable and variable environments, whilst unlearned behavior evolves in more constant conditions (Bergman & Feldman, 1995; Boyd & Richerson, 1985; Feldman et al., 1996; Stephens, 1991). This implies that the matters that humans communicate about change at a significantly faster rate than the focus of communication of other apes, and raises the question of what our ancestors needed to talk about that changed so quickly.

To my knowledge, there is no published hypothesis for the original function of language that meets all seven of these criteria. That is encouraging, as it implies that collectively they constitute a tough hurdle that any credible selective scenario for the evolution of language must overcome. Here, I present a plausible explanation that does pass these tests: language originally evolved to teach kin.

While social learning is widespread in animals, cumulative culture that ratchets up in complexity and diversity over time is unique to humans (Dean et al., 2012, 2014). Theoretical analyses have established that high-fidelity information transmission is necessary for cumulative culture (Lewis & Laland, 2012), and teaching is a prominent means by which high-fidelity transmission is achieved. I use the term ‘teaching’ broadly, referring not just explicit tutelage, or the teaching of skills, but also the passage of declarative knowledge that results in learning, and a host of more subtle processes, including the use of eye contact, joint reference and utterances to help pupils to learn to what they should pay attention, or to what a symbol refers (i.e. ‘pedagogical cueing’) (Tomasello, 1999; Gergely & Csibra, 2005; Gergely et al. 2007; Csibra 2010). Defined in this way, teaching is rare in nature, but universal in human societies (Tomasello, 1999; Gergely & Csibra, 2005; Gergely et al., 2007; Csibra, 2010). Mathematical analyses reveal stringent conditions that must be met for teaching to evolve, but show that cumulative culture relaxes these conditions (Fogarty et al., 2011). This implies that teaching and cumulative culture coevolved in our ancestors, creating for the first time in the history of life on earth a species that taught their relatives across a broad range of contexts. Further increments in the probability of teaching come when the costs of teaching are low or can be offset against the costs of provisioning, when teaching is highly accurate and effective in transmission, and when there is a strong degree of relatedness between tutor and pupil (Fogarty et al., 2011). Given that an animal is teaching, adaptations that reduce the costs of teaching without diminishing effectiveness, or that enhance effectiveness without increasing costs, ought to be favored by selection. Should a character appear that simultaneously both increases the effectiveness and reduces the costs of teaching, then we might envisage that it would be subject to strong positive selection, but, crucially, *only in a population of teachers*. The more teaching contexts in which the character could be applied, the greater its selective advantage.

Language is such a character. First, language is an extremely cheap way to teach. Telling someone where to find a food patch is far easier than taking them there. Instructing a child that the red berries are poisonous is more straightforward than getting this across through other means. A simple “yes” or “no”, or “this way, not that”, will allow a tutor to provide helpful guidance to a pupil acquiring a new skill at low cost. Second, language is an exceedingly accurate way to teach, bringing a precision to information transfer that is virtually impossible to achieve through other means. This precision, combined with the efficiency by which language allows tutors to cue their pupils onto relevant events as they occur, and to provide instructive guidance during skill learning, means that teaching through language greatly enhances knowledge transfer. Simple utterances that carry messages like “pay attention”, “dig here”, “like this”, “faster”, “this way”, provide invaluable clues, helping the learner to focus in on what actions need to be imitated, or where precisely new skills need to be applied. Once an individual is committed to teaching, language is by far and away the most efficient means to do so. That is why almost all teaching in human societies occurs through use of language (Hewlett et al., 2011; Tehrani & Riede, 2008). In addition, language extends the range of phenomena that can be taught, allowing knowledge of abstract concepts to be imparted, the understanding of which may significantly enhance the pupil's performance, as well as instruction about the past and future, and distant events or objects.

Theoretical analyses show that teaching is more likely to be advantageous amongst close relatives than unrelated individuals (Fogarty et al., 2011), and 2 million years ago our ancestors were living in small, kin-structured groups (Stringer & Andrews, 2005). Humans may have evolved as cooperative breeders (Hrdy, 1999), heavily reliant on ‘allomothering’ helpers (See also, Isler & van Schaik, 2012). The unusually long period of juvenile dependence in our species helps make the teaching of life skills to children economical, as the costs of instruction can be offset against years of feeding and caring. The quicker children can be taught to fend for themselves, the lower the burden of childcare. Recent thinking on the evolution of early *Homo* suggests that increases in brain size were coupled with increased tool making and stone transport, dietary expansion, and greater developmental plasticity (Anton, 2014). This means that there would be plenty to teach, as our hominin ancestors subsisted on a broad omnivorous diet, reliant on a large number of extractive foraging and tool-using skills (Stringer & Andrews, 2005; Anton, 2014). This period in human history was the dawn of cumulative culture, when our ancestors first began manufacturing stone tools, and using the flakes to butcher carcasses for food, and in other ways. Here, then, is a setting in which teaching amongst close relatives could be beneficial across a broad range of contexts. I submit that language originally evolved to enhance the efficiency and scope of this teaching. As a hypothesis, it sounds plausible enough, but does it meet the seven criteria?

Firstly, if language evolved to teach relatives (Fitch, 2004), we would expect it to be honest. No new conflicts of interest between signaller and receiver arise in a teaching scenario. The function of teaching is to ensure accurate knowledge transfer so that pupils can acquire fitness-enhancing skills and information. If pupils are relatives, deception or inaccuracy on the part of the tutor would undermine the indirect inclusive fitness benefits that teaching provides to the tutor (Fogarty et al., 2011). Provided early communication was initially restricted to that which was taught, the interests of transmitter and receiver are broadly aligned. Plausibly tutor and pupil (e.g. parents and offspring) might differ in how much instruction they would like to see imparted, but what teaching does occur is expected to be honest, as inaccurate instruction is simply wasting the tutor’s time and effort. Likewise, the cooperativeness of early language is readily understood: if language evolved to teach, it emerged in a context that was already cooperative. No difficulty arises in explaining why it benefits a tutor to impart valuable information to a pupil, if by doing so he or she teaches life skills to a relative in a manner that ultimately increase the tutor's inclusive fitness.

How language could have gotten started in a teaching context, and how symbols acquired their meanings (criteria 3 and 4) are also easily envisaged. Simple attention-grabbing commands would do little to get most messages across, but they have been proven to help to facilitate social learning. One of the challenges of imitation is that the demonstrator's behavior is a constant stream of actions, which means that what should be copied is not always apparent to the observer, nor where the relevant actions start and stop. Here, a simple verbal (or even non-verbal) cue can be invaluable. This has been demonstrated through developmental psychology experiments, where extensive data now show that adults cue the learning of babies and young children with simple vocalizations. Such cues are known to generate referential expectations in infants, triggering a tendency to follow the gaze of adults as they orientate, such as shifts in the adult’s gaze towards the particular objects with which the adult interacts, and facilitating joint attention (Tomasello, 1999; Gergely & Csibra, 2005; Gergely et al., 2007; Csibra, 2010). The use of such cues, and the resulting gaze following and joint attention, are thought to contribute to the infant learning about both the properties of objects, and how they can be manipulated, as well as the meaning of words (Tomasello, 1999; Gergely & Csibra, 2005; Gergely et al. 2007; Csibra; 2010). At the same time, pointing, gesture and movement can ground teaching utterances, to provide meaning to unfamiliar terms. A tutor can exclaim “here” at the same time as pointing to where the stone must be hit. He or she can mime digging with a stick whilst uttering the word “dig”. Utterances equivalent to “no, this way” can be emitted whilst manually shaping the pupil's body movements. Experimental findings demonstrate that this is not only plausible, but regularly happens when children learn new skills (Tomasello, 1999; Dean et al., 2012). Hence, in the context of teaching, how early language could have achieved symbol grounding, and how it could have been of value when comprising just a handful of words, becomes feasible to envisage.

The requirement that the theory should provide an explanation for language's power of generalization is also satisfied. Teaching through language, once started, could be applied to multiple difficult-to-learn proficiencies, including extractive foraging procedures, food-processing methods, and hunting skills. Paleontologists’ investigations suggest that the diet of *Homo* species was highly versatile, with our ancestors eating a broad range of foods, including fruits, tougher foods like woody plants, and various animal tissues (Stringer and Andrews, 2005). This broadening of the hominin diet is associated with increasing reliance on difficult-to-access but nutrient rich foodstuffs that required extraction from a substrate and some form of processing. Often, this food processing required not just tool use but prior technological manufacture. The teaching of foraging, hunting and scavenging methods, tool manufacture, food preparation and food-processing skills, fire maintenance, and collective defense, some of which required the coordinated actions of multiple individuals, would particularly have benefitted from verbal instruction. In this way, a modest protolanguage would be expected to become increasingly elaborate, and to generalize in a number of dimensions.

The uniqueness criterion is also met. No animals aside from humans (and perhaps their immediate ancestors) evolved language because humans alone were engaged in extensive teaching. In the absence of widespread teaching, no selection for language to reduce teaching costs or promote teaching efficiencies would occur. Only in hominins did language, teaching and cumulative culture coevolve.

With respect to the question of why early language needed to be learned, it is relevant to note that chimpanzees and orangutans both have extensive tool-using repertoires, as well as behavioral traditions that exhibit considerable inter-population variation (Whiten et al., 1999; van Schaik et al., 2003). Members of our genus are likely to have constructed richer and geographically more diverse cultural repertoires than contemporary apes, including tool-using and foraging traditions, a perspective supported by recent archaeological evidence (McBrearty & Brooks, 2000; d’Errico & Stringer, 2011). A shift from unlearned to learned vocalization suggests an increase in the rate of change of features of the environment that select for primate communication. However, an explanation based on independently changing external conditions, such as fluctuating climates, is not particularly compelling, both because the scale of climatic change is too slow, and because an external source of selection ought equally to have favored extensive learned communication in other primates. If, however, language initially evolved as an adaptation to cope with self-constructed elements of the environment, such problems are alleviated. From a comparative perspective, the most obvious features to fit the bill are cultural practices, particularly tool use, extractive foraging and material culture. Cultural practices are typically transmitted amongst close relatives, are deployed to exploit difficult-to-access but nutrient-rich foodstuffs, and are challenging to learn, making them precisely those traits that would benefit from teaching. At some stage in the last 2 million years, our ancestors began to generate cultural variants (e.g. tools, foraging techniques, social signals, courtship rituals, medicative treatments, gestures) at such a rate that they could no longer communicate about their world without being required to constantly update and elaborate communicative signals and meanings. If each new tool, or foraging technique, or display, or treatment has to be learned, and if, as the comparative evidence suggests, cultural variants such as tool use are typically learned by young apes from their mothers and older siblings (Whiten et al., 1999; Reader, 2000), then language may have coevolved with cultural complexity as a means of facilitating and enhancing socially transmitted life-skill acquisition in young hominins (Csibra & Gergely, 2011). Consistent with this hypothesis, a recent experimental archaeology study demonstrated that, across six measures, the transmission of stone tool making improves with teaching, and particularly with language, but not with imitation or emulation (Morgan et al., 2015).

Difficult though it is to be totally confident about any account of the selective scenario that originally favored language, the hypothesis that language originally evolved to teach, specifically, to teach close relatives, has many virtues. The account explains the honesty, cooperativeness, uniqueness and symbol grounding of language, as well as how language got started, its power of generalization, and why language is learned. The explanation meets all seven of the criteria required of a successful account of language origins, something that, to my knowledge, no other hypothesis has done. If I am correct, language is an adaptation that originally functioned to increase the accuracy, reduce the costs, and increase the scope of teaching.

Naturally, the selective scenario for language only begins here, and is likely to have been co-opted and amplified in a variety of ways. Fitch (2004) has argued that language originated to facilitate communication amongst close kin (Fitch, 2004, see also Nowicki & Searcy, 2014; Smit, 2014), and I endorse this position. In the kin-structured groups exhibited by our ancestors, early language, initially selected as an adjunct to the teaching of young by parents or siblings, could subsequently spread to teaching more distant relatives. This expansion would have been particularly relevant to activities such as collective foraging, scavenging and hunting, which required coordinated activity amongst multiple individuals. Here, the direct benefits of ensuring that relatives possess relevant skills and knowledge, in the form of enhanced foraging returns, would compensate for the reduction in the degree of relatedness amongst more distant relatives. Complex coordinated actions are often difficult to bring off without a means to teach, or tell, individuals what their specific roles should be. In this regard, language would prove an extremely powerful coordination tool (Sterelny, 2012).

Subsequently, with language, teaching could be extended to support other established cooperative processes, such as mutualistic exchanges, indirect reciprocity, and group selection (Fitch, 2005; Nowak & Highfield, 2011). I envisage a transition of early language from its origins in teaching kin to richer forms of language capable of supporting other forms of cooperation amongst non-kin (Laland, 2016). Both reciprocal altruism and mutualistic trade (at least, the trade of distinct, desired commodities) are surprisingly rare in other animals outside of the context of kinship (Fitch, 2005; Ridley, 2011). Trade seemingly requires some capacity to agree a rate of exchange, something that would be very difficult without at least proto-language, or through the flexible use of shared gesture. With the evolution of language, trade becomes a possibility, whilst with trade comes negotiation, and selection for still more developed communication (Pagel, 2012). Likewise, “for the mechanism of indirect reciprocity to work efficiently it needs gossip, from names to deeds and times and places, too” (Nowak & Highfield, 2011). Linguistically taught social norms allow humans to institutionalize the punishment of non-cooperative individuals, for instance, through policing or socially sanctioned retaliation, which theory and experimentation shows is a more effective means of preserving cooperation than individual-level punishment (Fehr & Gachter, 2002; Fehr & Fischbacher 2003), and potentially support cultural group selection (Boyd & Richerson, 1985; Henrich 2015). I agree with Pagel (2012) that “language evolved as a trait for promoting cooperation”, but differ in suggesting that the origins of language begin with a highly specific form of cooperation, namely teaching. Other cooperative contexts, for instance, reliant on reciprocity, trade and group selection, could certainly have exploited a pre-existing linguistic capability, generating selection for enhanced linguistic skills. Such selective feedback would likely have made a big difference both to the scale of human cooperation that ensued, and to the potency of human language (Pagel, 2012), plausibly helping to explain how early language extended into domains in which honesty could not be assumed, and vigilance against malevolence or incompetence was required. However, other cooperative contexts struggle to meet the *honesty* and *adaptive from outset* criteria described above, and hence cannot be how language got started.

Like others (Deacon, 1997; Bickerton, 2009), I suspect that our ancestors constructed a world sufficiently rich in symbolism to generate evolutionary feedback, in the form of self-modified selection pressures that favored structures in the mind that functioned to manipulate and use those symbols with efficiency (Rilling et al., 2008; Schenker et al., 2010; Erwin et al., 2010). This feedback is variously cast as manifestations of the Baldwin effect (Deacon, 1997) or of niche construction (Bickerton, 2009; Odling-Smee & Laland, 2009). Selection for more efficient and higher-fidelity forms of social learning has favored the evolution of specific structures and functional capabilities in the brain, in the process driving the evolution of brain and intelligence (Laland, 2016). The syntax that we witness in contemporary human language is only possible because of a long history, spanning perhaps 2 million years, of symbolic manipulation in protolanguage, which constructed selection pressures that, in turn, brought about significant changes in the hominin brain (Hauser, 1996; Bickerton, 2009).

As the sheer volume of symbols that our ancestors were required to learn the meaning of, and string together in unambiguous messages, increased, so it created the demand for rules and conventions specifying usage patterns (i.e. one important aspect of syntax). If words are simply strung together without syntax then ambiguities over their collective meaning rapidly arise, creating a heavy processing burden on the receiver. Syntax alleviates this burden by breaking up the message hierarchically and recursively into meaningful and readily comprehensible chunks, phrases and clauses, that the brain can easily and quickly process, and by introducing rules that eradicate ambiguities. With this syntax came not just full-blown language, but an almost infinite flexibility in usage. Words have highly restricted meanings until they are strung together, but in combination, underpinned by a mutually understood set of combinatorial rules, they are capable of communicating highly complex messages.

Language probably began as a means of reducing the costs of teaching complex foraging skills, but will have become coopted to teaching linguistic symbols too. Once early language itself became something that was frequently taught (although often implicitly, without overt tutelage), this in turn would have generated selection for effective means of teaching language to children, such as ‘infant-directed speech’ (‘motherese’) (Falk, 2004). Children are known to hear some linguistic structures selectively and to ignore others, a phenomenon that may have generated selection for language structure that is “child friendly” (Deacon, 1997; Falk, 2004; Fitch, 2004, 2005). Infant-directed speech is typically slower and higher in pitch than regular speech, and uses shorter and simpler words. Studies have shown that infants prefer to listen to this type of speech, that it is more effective in getting and infants’ attention, and that it helps infants to learn words faster, compared to standard speech (Thiessen et al., 2005; Fitch, 2010). The suggestion is commonly made that language learning by children is spontaneous, or ‘instinctive’ (Pinker, 1995), as if adults play little role. Such arguments underestimate the important ways in which adults facilitate language learning in children. Experimental studies show that the children who learn language fastest are those who receive the most acknowledgement and encouragement of what they say, who are given time and attention to speak, who are corrected, questioned, and spoken to in a child-friendly manner, and who are exposed to syntactically complex speech at the right time (Waterson, 1978; Huttenlocher et al., 2002; Thiessen et al., 2005; Fitch, 2010). Important elements of infant-directed speech, such as infants’ sensitivity to its linguistic features, or adults’ tendency to engage in behavior that elicits rewarding responses from infants (e.g. smiles), have been favoured through a biological evolutionary process.

As protolanguages began to increase in complexity from rudimentary foundations, they would have generated increasing strong selection for cognitive adaptations that facilitated language learning and transmission. For instance, compared to other primates, humans appear particularly adept at inferring the meaning of the utterances of others (Bloom, 1997, 2000; Fitch, 2010). While this ability is partly attributable to the aforementioned pedagogical activities of the transmitter, an enhanced capability to extract meaning through observation of others’ activities in the receiver is also likely. What is more, the selective feedback from symbolic manipulation to the human mind likely extends far beyond the acquisition of a capacity to extract meanings and comprehend syntax. Chomsky described language as the main engine of thought, and there is now little doubt that humans possess a mind uniquely fashioned to acquire and process information linguistically.

As the conventions of linguistic structure varied from one (proto)language to another, and changed over time, the rules of syntax would themselves have to be learned. Whether they are learned through a dedicated language acquisition device, as envisaged by Chomsky (1965), or through some more general process mechanisms, such as Bayesian learning, is a moot point. Either way, I envisage that the symbol-rich cultural world constructed by our ancestors was a major source of selection for enhancements in language learning. Language is itself a powerful example of niche construction (Bickerton, 2009; Odling-Smee & Laland, 2009), a dramatic change that our ancestors brought about in their (conceptual) world. Selective feedback from language would have operated at two levels, a gene-culture coevolutionary dynamic, where human cultural activities generate natural selection favoring enhanced language learning and transmission capabilities, but also a cultural evolution dynamic, whereby human cultural activities feed back to affect the learned properties of the language.

Brighton et al. (2005) labelled the latter process ‘cultural selection for learnability’. If linguistic structures are to persist over time they must repeatedly survive the process of being learned, expressed, and adopted by others. Children may appear pre-adapted to decipher the rules of syntax in part because languages have evolved to have rules that are easy to learn (Deacon, 1997; Brighton et al., 2005). The cultural evolution of language has been studied through mathematical modeling, and researchers have established that key properties of language, for instance compositionality, could evolve in this manner (Smith and Kirby, 2008; Kirby et al. 2007). Likewise, both transmission-chain experiments and mathematical models show how languages propagated culturally evolve in such a way as to maximize their own transmissibility, becoming easier to learn and more structured over time (Kirby et al., 2008). This research is important, as it shifts some of the explanatory burden for language away from natural selection for language-specific cognitive adaptations, and makes the challenge of explaining the origins of language more manageable (Smith and Kirby, 2008; Kirby et al., 2007, 2008, 2015).

I emphasize that the above account solely addresses the issue of the original function of language. I have said little to nothing about how vocal learning, generative computations underlying language, systems of semantic representations, phonological representations, or the interfaces between these, evolved, and nor have I explained how all of this internal machinery was externalized in linguistic communication, expressed acoustically or visually. I nonetheless hope that the account will prove of value by taking away a small part of the mystery of the origin of language.
